# LAMA2 regulates the fate commitment of mesenchymal stem cells via hedgehog signaling

**DOI:** 10.1186/s13287-020-01631-9

**Published:** 2020-03-25

**Authors:** Yuan Zhu, Xiao Zhang, Ranli Gu, Xuenan Liu, Siyi Wang, Dandan Xia, Zheng Li, Xiaomin Lian, Ping Zhang, Yunsong Liu, Yongsheng Zhou

**Affiliations:** 1 Department of Prosthodontics, Peking University School and Hospital of Stomatology, National Laboratory for Digital and Material Technology of Stomatology, Beijing Key Laboratory of Digital Stomatology, National Clinical Research Center for Oral Diseases, 22 Zhongguancun South Avenue, Beijing, 100081 People’s Republic of China; 2 Department of Materials Science and Engineering, College of Engineering, Peking University, Beijing, 100871 People’s Republic of China

**Keywords:** LAMA2, Osteogenic differentiation, Human mesenchymal stem cells, Hedgehog signaling

## Abstract

**Background:**

Bone defects are a common clinical condition that has gained an increasing amount of attention in recent years. Causes of bone defect include tumors, inflammation, and fractures. Bone tissue engineering is a novel treatment of bone defect, and human mesenchymal stem cells (hMSCs) are the ideal seed cells for bone tissue engineering due to their multi-lineage differentiation potential and immunogenicity. The laminin α2 (*LAMA2*) gene encodes the α2 subunit of laminins. Mutations in this gene have been reported to cause muscular dystrophy, but thus far no studies have elucidated the role of LAMA2 in the fate choices of MSCs. Here, we aimed to investigate the critical role of LAMA2 in the osteogenesis and adipogenesis of mesenchymal stem cells (MSCs).

**Methods:**

We investigated LAMA2 function in osteogenic and adipogenic differentiation of MSCs in vitro and in vivo through loss- and gain-of-function experiments. In addition, molecular mechanism was clarified by Western blot and siRNA.

**Results:**

Our results demonstrated that LAMA2 was a critical regulator for fate commitment of MSCs. Both in vitro and in vivo studies indicate that LAMA2 inhibits osteogenesis and promotes adipogenesis*.* Mechanistically, we found that LAMA2 regulated osteogenesis and adipogenesis of MSCs by modulating the hedgehog signaling pathway.

**Conclusions:**

The present work confirms that LAMA2 is a new molecular target for MSC-based bone regeneration.

## Background

Many diseases can cause bone defects, and the presence of bone defects leads to a poor quality of life. Recent studies have illustrated bone tissue engineering as a prospective therapeutic approach for bone regeneration [1, 2]. Seed cells, growth factors, and biological scaffolds are the three main factors of tissue engineering. Mesenchymal stem cells (MSCs) are the current focus of researchers as they can self-regenerate, have multidirectional differentiation ability, and are easy to obtain; therefore, they are a good candidate for use as seed cells in bone tissue engineering and have promising clinical prospects.

Many studies have demonstrated that MSCs can differentiate into osteoblasts, chondrocytes, and adipocytes [3, 4]. These findings have significantly promoted research into tissue engineering [5–7]. The balance between osteoblast and adipocyte formation is related to metabolic homeostasis [8, 9]. Many studies have shown that adipogenesis and osteogenesis are antagonistic to each other [10–13], but the underlying mechanisms remain largely unknown. Once the balance between osteogenesis and adipogenesis is broken, it would lead to bone metabolic diseases and even bone defects [14, 15]. Therefore, investigations of the molecular mechanism of osteogenic and adipogenic differentiation of MSCs would help in developing MSC-based treatment strategies for bone loss.

Laminins are composed of three subunits, named α, β, and γ. They regulate cell growth, movement, and attachment and participate in the formation of the basement membrane and attach to other proteins in the muscle cell. The *LAMA2* gene encodes the α2 subunit of laminins, and mutations in this gene have been shown to cause LAMA2-related muscular dystrophy [16, 17]. A study showed that laminin could regulate the osteogenic differentiation of dental follicle cells, and LAMA2 was found to be upregulated in dexamethasone-induced dental follicle cells [18]. However, it remains unclear how LAMA2 regulates the fate choices of MSCs. Therefore, we aimed to investigate the critical role of LAMA2 in the osteogenesis and adipogenesis of MSCs in this study. To this end, we studied the function of LAMA2 in osteogenic and adipogenic differentiation of MSCs in vitro and in vivo through loss- and gain-of-function experiments. The molecular mechanisms were studied by small interfering RNAs (siRNA) and Western blot analyses.

## Methods

### Culture, osteogenic induction, and adipogenic induction of MSCs

Primary human adipose-derived stem cells (hASCs) and human bone marrow mesenchymal stem cells (hBMMSCs) were obtained from ScienCell (San Diego, CA, USA) and cultured in Dulbecco’s modified Eagle’s medium (DMEM) or α-minimum essential medium (α-MEM). The proliferation medium (PM) contained 10% (v*/*v) fetal bovine serum (FBS) and 1% (v/v) antibiotics. The osteogenic medium (OM) contained 10% (v*/*v) FBS, 1% (v/v) antibiotics, 10 nM dexamethasone, 200 μM ascorbic acid, and 10 mM β-glycerophosphate. The adipogenic medium (AM) contained 10% (v*/*v) FBS, 1% (v/v) antibiotics, 50 nM insulin, 100 nM dexamethasone, 500 μM 3-isobutyl-1-methylxanthine, and 200 μM indomecin.

### Lentivirus infection

Lentiviruses targeting *LAMA2* (sh*LAMA2*-1 and sh*LAMA2*-2), negative control vectors (NC), and lentiviruses that segmentally express the *LAMA2* gene were purchased from GenePhama Co. (Suzhou, China). The sequences were as follows: sh*LAMA2*-1, GCCTGAGATTTCAGAGGATCC, and sh*LAMA2*-2, GCTCCCTATCTGGGAAACAAA. First, hASCs and hBMMSCs were cultured to 50% confluence and transfected in the presence of 5 μg/mL polybrene to promote transfection for 24 h. The lentivirus carried the plasmid vector containing fluorescence proteins and the puromycin resistance gene. Thus, stably transfected cells were detected by the addition of puromycin after incubation for 72 h.

### RNA interference

siRNAs targeting *GLI2* and NC were obtained from GenePharma, the sequences were presented in Additional file 1: Table S1. Cells were transfected with siRNAs using Lipofectamine 3000 (Invitrogen) and harvested for RNA and protein analyses after 48 h. For osteogenic and adipogenic differentiation, cells were transfected every 5 days in OM or AM and harvested after 7 or 21 days.

### Alkaline phosphatase (ALP) staining and ALP activity, Alizarin Red S (ARS) staining and quantification

The cells were divided into PM and OM culture groups. ALP staining was performed using NBT/BCIP staining kit (CoWin Biotech, Beijing, China) on day 7 after osteogenic induction. ALP activity was quantified using ALP assay kit (Nanjing Jiancheng Bioengineering Institute). Absorbance was measured at 520 nm, and the ALP activity was calculated.

On day 14 after osteogenic induction, 2% Alizarin Red buffer (Sigma-Aldrich) was used to stain cells. To quantify mineral accumulation, 100 mM of cetylpyridine solution was added to the wells of a multiwell plate. Mineral accumulation was quantified by measuring the absorbance at 562 nm after completely dissolving the cells.

### Oil Red O staining and quantification

Cells were inoculated and cultured in PM and AM separately, and Oil Red O staining and quantitative assessment were carried out on day 21 after adipogenic induction. The cells were fixed with 10% neutral formalin and then rinsed with 60% isopropanol. Cells were stained with Oil Red O working solution and observed under a microscope. For quantitative assessment, 100% isopropanol was added to each well containing the stained cells, after which absorbance was measured at 500 nm.

### Real-time quantitative PCR (RT-qPCR)

Total cellular RNA was extracted from MSCs cultured in proliferation or differentiation medium for 7 and 14 days with TRIzol reagent (Invitrogen, Carlsbad, CA, USA). PrimeScript RT Reagent Kit (Takara, Tokyo, Japan) was used to synthesize the cDNA. RT-qPCR was conducted with SYBR Green Master Mix on an ABI Prism 7500 real-time PCR System. Gene expression was normalized to the expression of *GAPDH*, which was used as the reference gene. The primer sequences of human *GAPDH*, *RUNX2*, *BGLAP*, *PPARγ*, *C/EBPα*, and *LAMA2* and mouse *Gapdh*, *Runx2*, *Pparγ*, and *Lama2* were presented in Additional file 1: Table S1.

### Western blot analysis

For detection of proteins, the cells were lysed in lysis buffer containing 2% proteinase inhibitor. Proteins extracts were subjected to 5% SDS-PAGE and transferred to polyvinylidene fluoride membrane. The membrane was incubated with the primary antibodies overnight; then, it was incubated with peroxidase-conjugated secondary antibodies at room temperature. The visualized immunoreactive protein bands were detected using an enhanced chemiluminescence (ECL) kit (CWBIO, Beijing, China).

### In vivo implantation of MSCs, ectopic bone, and ectopic adipose tissue formation

Lenti-NC-, Lenti-sh*LAMA2*-1-, and Lenti-sh*LAMA2*-2-transfected MSCs (P4) were cultured in PM for 1 week. The resultant cells were used for MSCs/β-TCP (RB-SK-005G) composite scaffold construction and implantation in nude mice. After trypsinization and resuspending, 1 × 10^6^ cells from each of the three groups were inoculated into cryotubes containing β-TCP powder (approximately 3 × 2 × 2 mm^3^).

Lenti-NC-, Lenti-sh*LAMA2*-1-, and Lenti-sh*LAMA2*-2-transfected MSCs (P4) were cultured in AM for 1 week. 1 × 10^6^ cells from each group were trypsinized and resuspended separately, mixed with the collagen membrane scaffold material (approximately 8 × 8 × 2 mm^3^ per tube), and placed in a cryotube.

Then, the mixtures (*n* = 10 per group) were placed in a shaker at 37 °C for 1 h and then centrifuged at 150*g* for 5 min to allow the cells to adhere to the β-TCP or collagen membrane scaffold. The mixtures were then implanted into female BALB/c nude mice. Eight weeks after implantation, the ectopic bone-like tissues were harvested and analyzed by hematoxylin and eosin (H&E), Masson’s trichrome, and immunohistochemical (IHC) staining. Adipose tissues were harvested after 6 weeks and the tissues were sectioned and analyzed by staining with H&E and Oil Red O.

### Micro-computed tomography (micro-CT) analyses of mice

Eight-week-old mice (10 per group) underwent sham or ovarian surgery and were euthanized 3 months after the surgery. Femur samples were scanned using the Inveon MM System (Siemens) micro-CT. The scanning conditions were 60 kV, 500 μA, and precision 8.82 μm. Parametric analysis was performed using Inveon Research Workplace (Siemens) software. The analysis area was 0.5–1 mm proximal to the epiphysis. The parameters analyzed included bone volume/total volume (BV/TV), and trabecular thickness (Tb.Th), number of trabecular bone (Tb.N), and trabecular space (Tb.Sp).

### Statistical analysis

SPSS 19.0 software was used for the statistical analysis. Data were expressed as means ± standard deviation. Differences between the two groups were analyzed by the independent two-tailed Student’s *t* tests. One-way ANOVA and Tukey’s post hoc test were used for comparison between groups. Values of *p* < 0.05 were considered statistically significant.

## Results

### LAMA2 is involved in MSCs cell fate determination

**Fig. 1:**
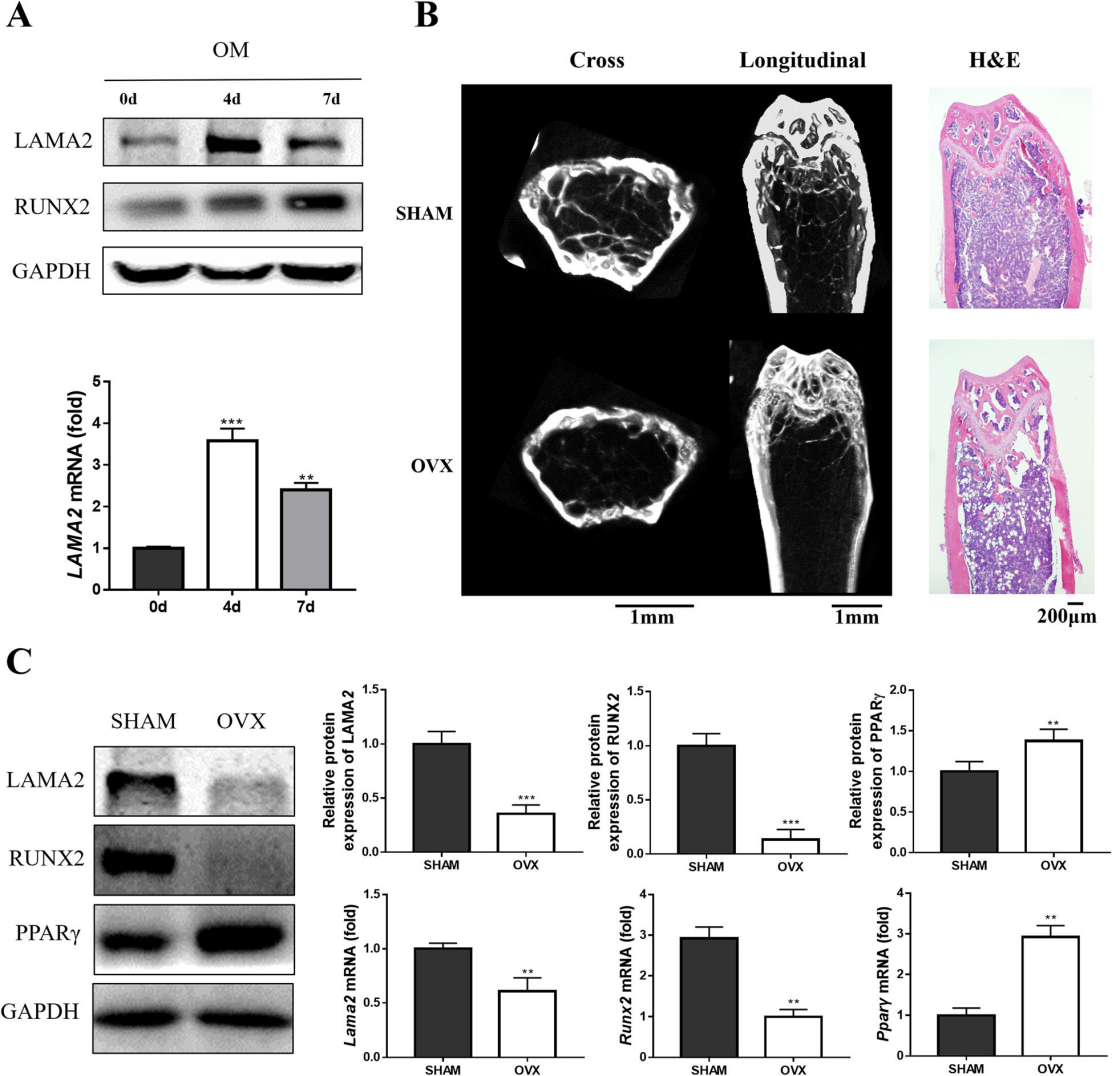
LAMA2 is a potential target for osteoporosis treatment. **a** LAMA2 expression during the osteogenic differentiation determined by Western blot and RT-qPCR. **b** Representative micro-CT and H&E staining of bone loss in OVX mice. **c***Lama2*, *Runx2*, and *Pparγ* mRNA expression levels and LAMA2, RUNX2, and PPARγ protein levels in bone marrow-derived MSCs from OVX mice and sham mice. **p < 0.01, ***p < 0.001

We studied the expression profile of LAMA2 to determine its involvement in osteogenic differentiation. LAMA2 expression initially increased during osteogenic differentiation and subsequently decreased (Fig. 1A). Then we investigate the status of LAMA2 in mice bone marrow mesenchymal stem cells. The results of the micro-CT scan and H&E staining showed a massive loss of trabecular bone in ovariectomy (OVX) mice (Fig. 1b and Additional file 2: Figure S1). Analysis of the LAMA2 mRNA and protein expression levels in BMMSCs revealed significantly decreased expression in OVX mice compared with sham mice (Fig. 1c).

### LAMA2 inhibited osteogenic differentiation of MSCs in vitro

**Fig. 2:**
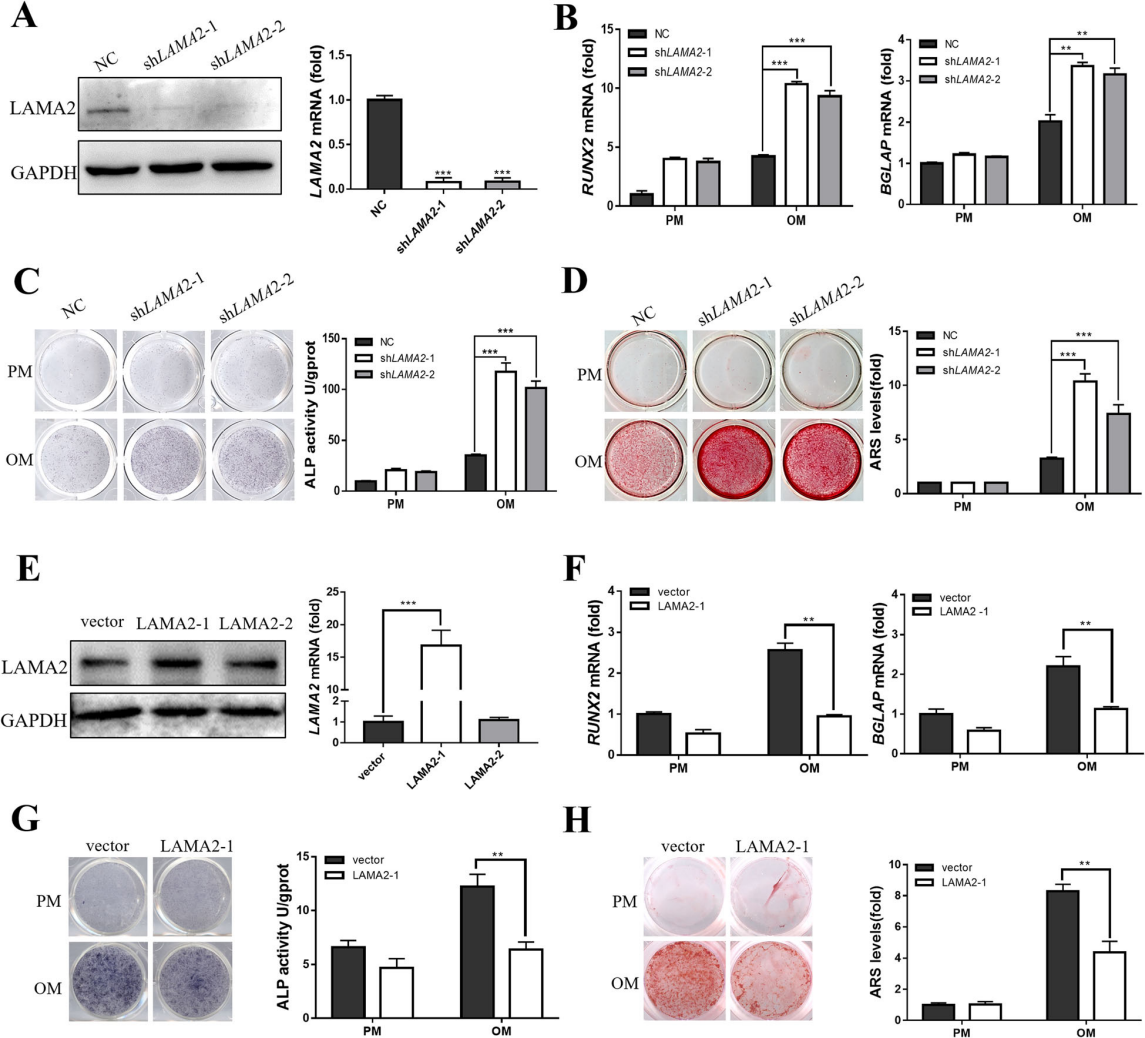
LAMA2 inhibited osteogenic differentiation of MSCs in vitro. **a** LAMA2 expression detected by Western blot and RT-qPCR. **b** RT-qPCR results revealed that *LAMA2* suppression enhanced the mRNA expression of *RUNX2* and *BGLAP*. **c***LAMA2* knockdown enhanced osteogenesis, as indicated by ALP staining (left) and quantification (right) in MSCs. **d***LAMA2* knockdown enhanced ARS staining (left) and quantification (right). **e** LAMA2 expression detected by Western blot and RT-qPCR. **f***LAMA2* overexpression reduced the mRNA expression of *RUNX2* and *BGLAP* detected by RT-qPCR. **g***LAMA2* overexpression reduced osteogenesis as indicated by ALP staining (left) and quantification (right) in MSCs. **h***LAMA2* overexpression reduced ARS staining (left) and quantification (right). **p < 0.01, ***p < 0.001

We established *LAMA2* knockdown MSCs to further study the effects of LAMA2 on osteogenic differentiation. Transfection efficiency was confirmed by fluorescence microscopy (Additional file 3: Figure S2). We also analyzed the mRNA expression of *LAMA2*, and the *LAMA2* knockdown groups (sh*LAMA2*-1 and sh*LAMA2*-2) exhibited a 90% decrease in expression compared with the NC group (Fig. 2a). Consistent with these findings, the results of the Western blot analysis also showed a significant decrease in the protein levels in the *LAMA2* knockdown cells (Fig. 2a). Cells from the *LAMA2* knockdown and control groups were incubated with either basal medium or osteogenic induction medium. *RUNX2* and *BGLAP* expressions were detected on days 7 and 14, respectively. *LAMA2* knockdown led to enhanced mRNA expression of *RUNX2* and *BGLAP* (Fig. 2b)*.* In addition, *LAMA2* knockdown significantly enhanced osteogenesis, as indicated by ALP staining and quantification (Fig. 2c). Similar results were also observed following ARS staining and quantification (Fig. 2d).

Next, we infected MSCs using fragments of the ectopic *LAMA2* lentivirus (due to its long sequence and large molecular weight, we divided the LAMA2 protein into two fragments) and verified *LAMA2* overexpression by RT-qPCR and Western blot analysis (Fig. 2e), the results showed that the cells infected by the first fragment could overexpress LAMA2 significantly. The results of the RT-qPCR analysis showed that *RUNX2* and *BGLAP* expression levels were significantly decreased in *LAMA2*-overexpressing cells after osteogenic induction (Fig. 2f). Moreover, *LAMA2* overexpression led to decreased staining with ALP (Fig. 2g), and ARS staining showed decreased mineralization in the MSCs overexpressing *LAMA2* (Fig. 2h).

### LAMA2 inhibited osteogenic differentiation of MSCs in vivo

**Fig. 3:**
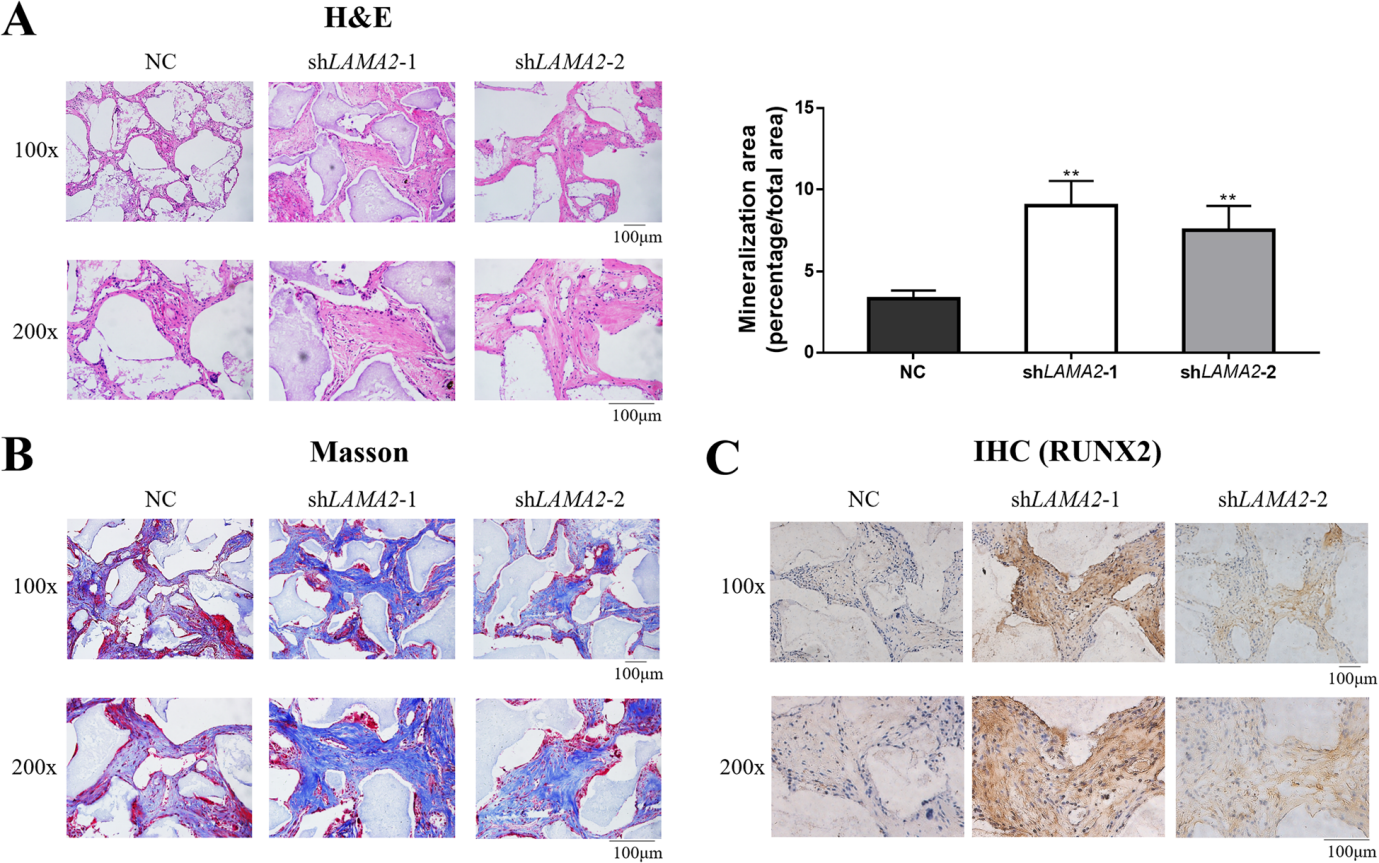
LAMA2 inhibited osteogenic differentiation of MSCs in vivo. **a** The neo-generated tissues were characterized by H&E staining and showed newly formed bone-like tissue in the sh*LAMA2* groups. The area of bone formation of sh*LAMA2* groups were more than the NC group detected by quantitative measurements of bone-like tissues. **b** Masson’s staining of histological sections. **c** IHC staining of RUNX2. **p < 0.01

To further determine the role of LAMA2 in osteogenic differentiation, nude mice were implanted with MSCs stably expressing sh*LAMA2* and control cells mixed with β-TCP. The neo-generated tissues were collected for 8 weeks after implantation. Progressive tissue development was further characterized by H&E staining. The area of bone formation was larger in the sh*LAMA2* groups than in the NC group, as shown by quantitative measurements of bone-like tissues (Fig. 3a). In addition, *LAMA2* knockdown groups had more bone tissue-like constructs as shown by Masson’s trichrome staining and IHC staining of RUNX2 (Fig. 3b, c).

### LAMA2 promoted adipogenic differentiation of MSCs

**Fig. 4:**
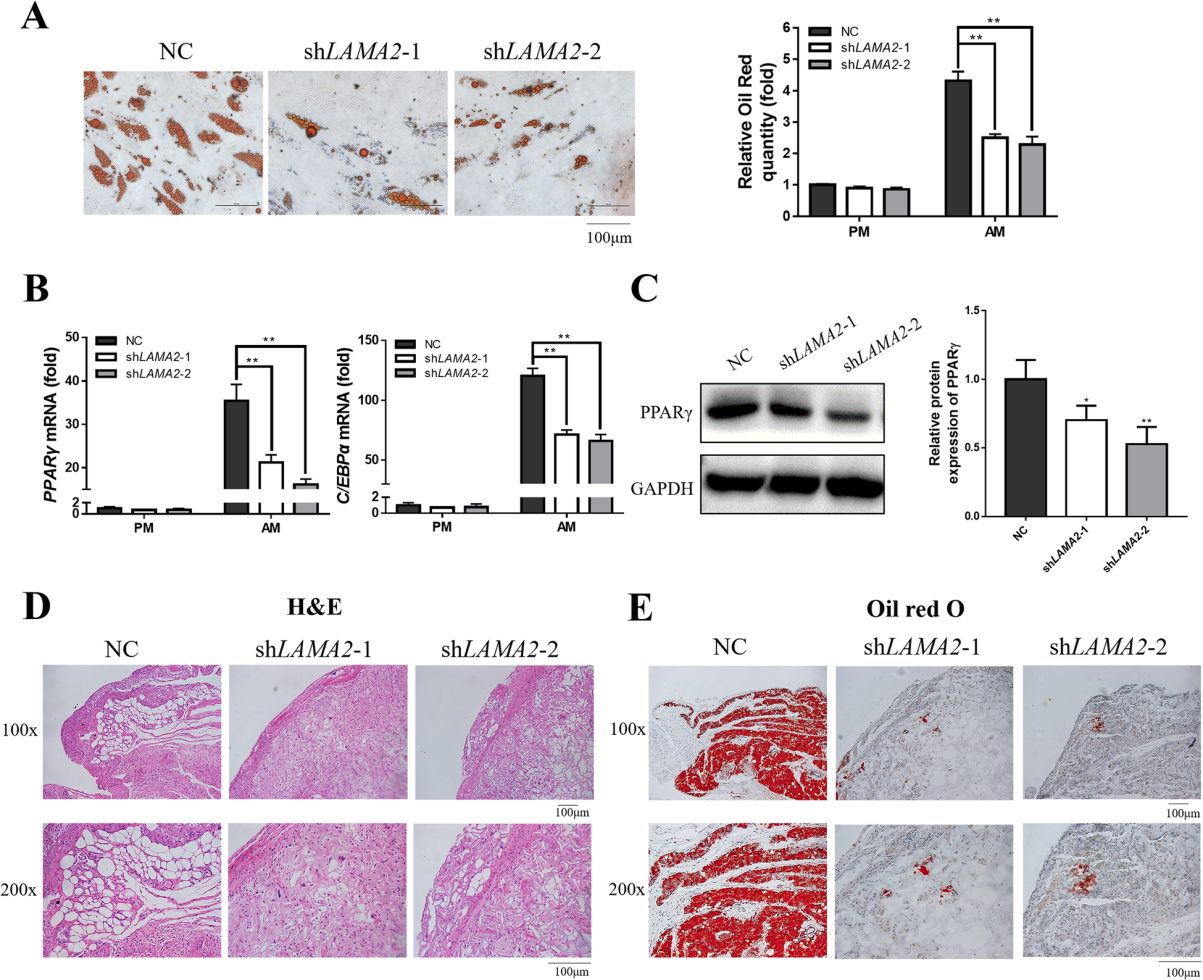
LAMA2 promoted adipogenic differentiation of MSCs in vitro and in vivo. **a** Knockdown of *LAMA2* inhibited adipogenesis as indicated by Oil Red O staining. **b** Western blot (left) showed that the protein expression of PPARγ was upregulated in the *LAMA2* knockdown group compared with the control group (NC) after 21 days of adipogenic induction. Western blot quantification (right) showed the same result. **c** Suppressing the expression of *LAMA2* inhibited the mRNA expression of *PPARγ* and *C/EBPα*, as detected by RT-qPCR. **d** H&E staining illustrated that *LAMA2* knockdown groups had fewer adipose tissue-like constructs in vivo. **e** Oil Red O staining illustrated that *LAMA2* knockdown groups had fewer adipose tissue-like constructs in vivo. *p < 0.05, **p < 0.01

To further investigate the potential effect of LAMA2 during adipogenic differentiation of MSCs, *LAMA2* knockdown MSCs were examined by Oil Red O staining after adipogenic induction. As shown in Fig. 4a, Oil Red O staining and quantification of *LAMA2* knockdown cells showed significantly fewer lipid droplets compared with control cells. In addition, both *PPARγ* and *C/EBPα* expression levels in the control group were significantly higher than in the *LAMA2* knockdown group (Fig. 4b). Moreover, Western blot analysis showed that PPARγ protein expression was downregulated during adipogenesis in *LAMA2* knockdown MSCs (Fig. 4c). To further detect the effect of *LAMA2* in adipogenic differentiation in vivo, we combined three MSCs groups (sh*LAMA2*-1, sh*LAMA2*-2, and NC) with a collagen sponge, and then implanted them into nude mice. The mixtures were collected after implantation for 6 weeks. Both H&E and Oil Red O staining results revealed fewer adipose tissue-like structures in the *LAMA2* knockdown groups (Fig. 4d, e).

### LAMA2 regulated the osteogenic and adipogenic differentiation of MSCs via hedgehog signaling pathway

To clarify the mechanism of LAMA2 regulating osteogenic and adipogenic differentiation, we screened key factors related to osteogenic and adipogenic differentiation and detected that hedgehog signaling was involved in the LAMA2-regulated osteogenic differentiation of MSCs.

**Fig. 5:**
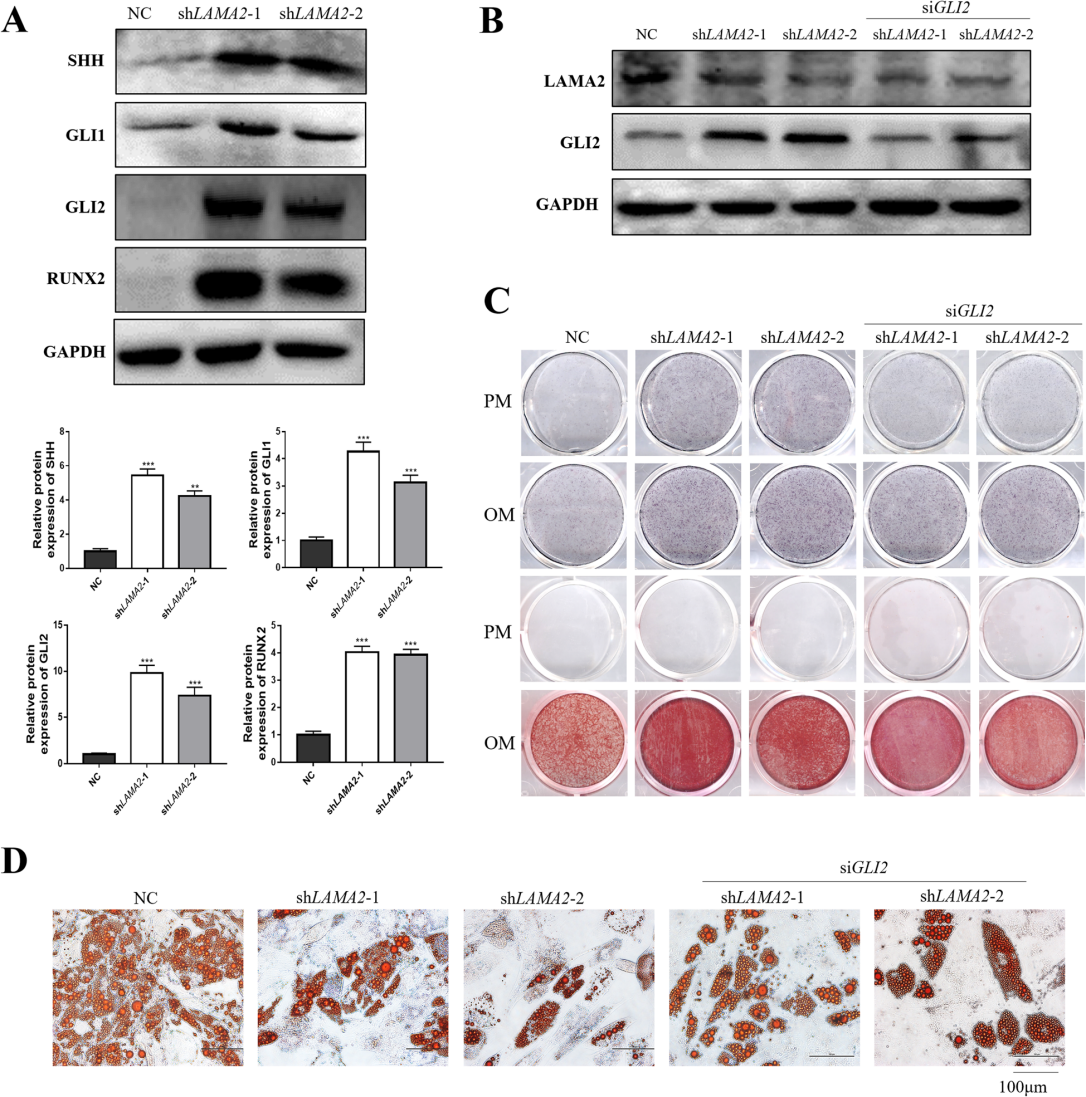
LAMA2 regulated osteogenic differentiation of MSCs via hedgehog signaling pathway. **a***LAMA2* knockdown enhanced the expression of SHH, GLI1, GLI2, and RUNX2. **b** Double knockdown of *LAMA2* and *GLI2* validated by Western blot analysis. **c** ALP and ARS staining in the control, *LAMA2* knockdown, and *LAMA2/GLI2* knockdown groups. **d** Oil Red O staining in the control, *LAMA2* knockdown, and *LAMA2/GLI2* knockdown groups. **p < 0.01, ***p < 0.001

It was reported that the enhancement of the hedgehog pathway increased osteogenesis and inhibited adipogenesis [19–21]. We detected a dramatic increase in the expression of SHH, GLI1, and GLI2 of the hedgehog signaling pathway in *LAMA2* knockdown cells (Fig. 5a). To further clarify LAMA2 regulation of osteogenesis and adipogenesis via the hedgehog pathway, we produced si*GLI2* in MSCs to block the hedgehog pathway, and RT-qPCR and Western blot analysis were used to verify the efficiency of *GLI2* silencing (Additional file 4: Figure S3). Then we established *LAMA2* and *GLI2* double knockdown cells and used Western blot analysis to verify the efficiency of double knockdown of *LAMA2* and *GLI2* (Fig. 5b). ALP and ARS staining showed that the double knockdown of *LAMA2* and *GLI2* inhibited the promoting effect of *LAMA2* knockdown on osteogenesis (Fig. 5c). Moreover, the depressed adipogenic differentiation by *LAMA2* knockdown was rescued in these double knockdown cells (Fig. 5d). Based on these results, our data suggest that LAMA2 regulates osteogenesis and adipogenesis by regulating the hedgehog signaling pathway.

## Discussion

In this study, we detected the crucial role of LAMA2 in the fate choices of MSCs. *LAMA2* inhibition was found to promote osteogenic differentiation and inhibit adipogenic differentiation both in vitro and in vivo. Furthermore, *LAMA2* overexpression could effectively suppress the osteogenic differentiation of MSCs.

Bone homeostasis depends on the resorption and formation of bones. Osteoporosis is caused by enhanced bone resorption and suppressed bone formation. Osteoporosis animal model is commonly used to study bone metabolism disorders and bone regeneration model, and OVX mice are a common osteoporosis model [22–24]. During the development of osteoporosis, BMMSCs have been shown to exhibit an increased number of adipogenic cells and a decline in the number of osteoblastic cells [25, 26]. It was worth noting that LAMA2 expression was reduced in OVX mice, but it increased first and then decreased during osteogenic differentiation. It was reasonable to assume that LAMA2 was degraded to prevent over-differentiation after early promotion of osteogenic differentiation.

Laminins are major basement membrane proteins consisting of alpha, beta, and gamma chains, and the *LAMA1–5* genes encode laminin alpha 1–5 chains, respectively. Diseases associated with LAMA1 include the Poretti-Boltshauser syndrome and cerebellar dysplasia with cysts [27, 28]. The *LAMA2* gene encodes the laminin alpha 2 chain, and mutations in this gene are thought to be responsible for merosin-deficient congenital muscular dystrophy [29]. LAMA3-related diseases include laryngeal cartilage skin syndrome and epidermolysis bullosa [30, 31], LAMA4-related diseases include cardiomyopathy [32], while LAMA5-related diseases include peripheral retinal degeneration and nephrotic syndrome [33, 34]. Several studies have shown that LAMA4 is closely related to adipogenesis. Vaicik et al. found that LAMA4 could affect adipose tissue expansion and function [35, 36], and Yamashita et al. found that laminin α4 chain fragment inhibited adipogenesis [37]. *LAMA4* is located on chromosome 6q21 while *LAMA2* is located on 6q22-q23 [38], so we want to further study whether *LAMA2* plays a similar role as *LAMA4* in adipogenesis. Some studies found an antagonistic relationship between the osteogenic and adipogenic differentiation of MSCs [39–41]. There is a balance between osteogenesis and adipogenesis, promoting the osteogenic differentiation and inhibiting the adipogenic differentiation of MSCs are the key directions in which bone regeneration progresses in bone tissue engineering. However, no studies have reported the effects of LAMA2 on osteogenesis and adipogenesis.

With an increase in age, the amount of adipose tissue increases while the amount of cancellous bone decreases. This is related to changes in the relative signaling pathways, which increases the differentiation of MSCs into adipocytes and decreases the differentiation of osteoblasts [8, 9, 42]. In this study, we found that LAMA2 regulates osteogenesis and adipogenesis of MSCs via modulating the hedgehog signaling pathway. The hedgehog signaling pathway is involved in bone formation and in osteogenic and adipogenic differentiation of MSCs which indicates that targeted regulation of hedgehog signaling is a potential target for the treatment of bone-related diseases such as osteoporosis and fracture healing [19, 20, 43–47].

Collectively, our research has some limitations. First, we did not determine the effects of LAMA2 overexpression on osteogenesis and adipogenesis in vivo. Second, we did not use a knockout mouse model in this study. Further studies on LAMA2 in a knockout mouse model investigating the effects on osteogenesis and adipogenesis should be performed.

## Conclusions

This is the first study to demonstrate that LAMA2 is a negative regulator of osteogenesis and a positive modulator of adipogenesis both in vitro and in vivo via hedgehog signaling. This work pointed out a new important function of LAMA2 and clarified its underlying molecular mechanism of a directional way that could be used as a novel molecular target in bone tissue engineering.

## Supplementary information

**Additional file 1: Table S1.** Sequences of RNA and DNA oligonucleotides.

**Additional file 2: Figure S1.** Bone volume, trabecular number, trabecular spacing, and trabecular thickness detected in Sham and OVX mice.

**Additional file 3: Figure S2.** Microscopic images of green fluorescence protein (GFP)-positive MSCs under ordinary and fluorescent light.

**Additional file 4: Figure S3.** RT-qPCR and Western blot analysis were used to verify the efficiency of GLI2 silencing in MSCs.

## Data Availability

The authors confirm that all data underlying the findings are fully available.

## Abbreviations

AM: Adipogenic medium
ARS: Alizarin Red S
BGLAP: Bone gamma-carboxyglutamic acid-containing protein
BMMSCs: Bone marrow mesenchymal stem cells
BV/TV: Trabecular bone volume/tissue volume
C/EBPα: CCAAT/enhancer-binding protein α
FBS: Fetal bovine serum
GAPDH: Glyceraldehyde3-phosphate dehydrogenase
GLI1: Glioma-associated oncogene family zinc finger 1
GLI2: Glioma-associated oncogene family zinc finger 2
H&E: Hematoxylin and eosin
hASCs: Human adipose-derived stem cells
hBMMSCs: Human bone marrow mesenchymal stem cells
hMSCs: Human mesenchymal stem cells
IHC: Immunohistochemical
LAMA2: Laminin α2
LP: Alkaline phosphatase
Micro-CT: Micro-computed tomography
MSCs: Mesenchymal stem cells
OM: Osteogenic medium
OVX: Ovariectomy
PM: Proliferation medium
PPARγ: Peroxisome proliferator-activated receptor γ
RT-qPCR: Real-time quantitative PCR
RUNX2: Runt-related transcription factor 2
SDS-PAGE: Sodium dodecyl sulfate polyacrylamide gel electrophoresis
SHH: Sonic hedgehog signaling molecule
siRNA: Small interfering RNA
Tb.N: Trabecular number
Tb.Sp: Trabecular spacing
Tb.Th: Trabecular thickness
β-TCP: β-Tricalcium phosphate
