# Supplementary material for: LAMA2 regulates the fate commitment of mesenchymal stem cells via hedgehog signaling
Source: Stem Cell Res Ther. 2020 Mar 25;11:135. doi: 10.1186/s13287-020-01631-9 (PMC7093965; doi:10.1186/s13287-020-01631-9)
Supplement: Supplementary file 1 — Additional file 1: Table S1. Sequences of RNA and DNA oligonucleotides. [file 13287_2020_1631_MOESM1_ESM.docx]

**Table 1** Sequences of RNA and DNA oligonucleotides

| **Name** | **Sense Strand/Sense Primer (5'-3')** | **Antisense Strand/Antisense Primer (5'-3')** |  |
| --- | --- | --- | --- |
| **siRNA** | | | |
| *GLI2* | GUGACACCAACCAGAACAATT | UUGUUCUGGUUGGUGUCACTT |  |
| NC | UUCUCCGAACGUGUCACGUTT | ACGUGACACGUUCGGAGAATT |  |
| **Primers for RT-qPCR** | | | |
| *LAMA2* | ACGCTGAGAAACTTGAGGCCA | TCAATATCTTTGAGGCAGCCGGA |  |
| *BGLAP* | AGCAAAGGTGCAGCCTTTGT | GCGCCTGGGTCTCTTCACT |  |
| *RUNX2* | CCGCCTCAGTGATTTAGGGC | GGGTCTGTAATCTGACTCTGTCC |  |
| *PPARγ* | GAGGAGCCTAAGGTAAGGAG | GTCATTTCGTTAAAGGCTGA |  |
| *C/EBPα* | CGCAAGAGCCGAGATAAAGC | CACGGCTCAGCTGTTCCA |  |
| *GAPDH* | GAAGGTGAAGGTCGGAGTC | GAAGATGGTGATGGGATTTC |  |
| *Lama2* | GACAGCGTGGCCAAAACGAA | AGTGCCTGCATCTGCAATGAT |  |
| *Runx2* | TCCACCACGCCGCTGTCT | TCAGTGAGGGATGAATGCT |  |
| *Pparγ* | TGTGAGACCAACAGCCTGAC | AAGTTGGTGGGCCAGAATGG |  |
| *Gapdh* | TGGAGTCTACTGGCGTCTT | TGTCATATTTCTCGTGGTTCA |  |
