# Supplementary figures and images for: LAMA2 regulates the fate commitment of mesenchymal stem cells via hedgehog signaling
Source: Stem Cell Res Ther. 2020 Mar 25;11:135. doi: 10.1186/s13287-020-01631-9 (PMC7093965; doi:10.1186/s13287-020-01631-9)

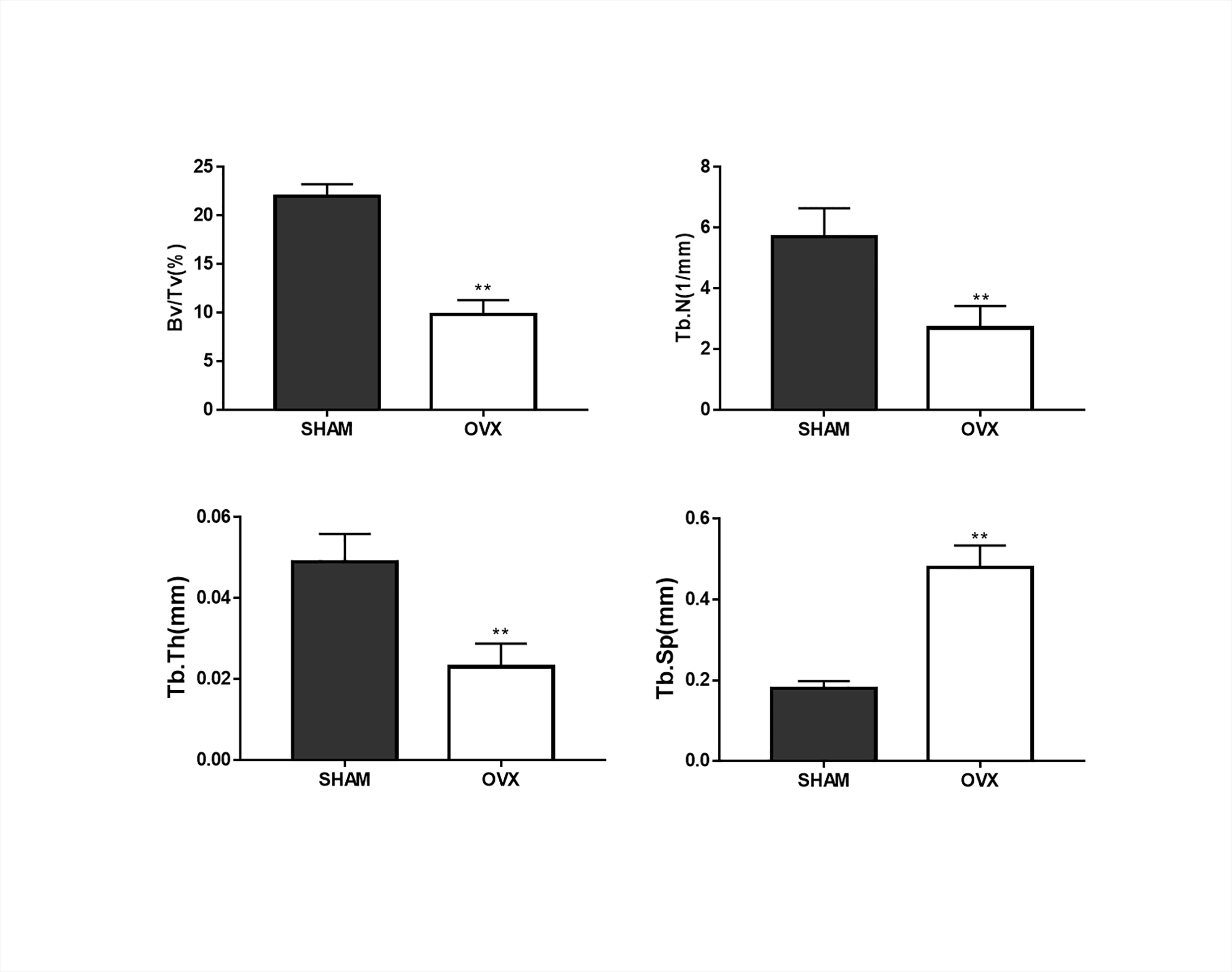

Supplement: Supplementary file 2 — Additional file 2: Figure S1. Bone volume, trabecular number, trabecular spacing, and trabecular thickness detected in Sham and OVX mice. [file 13287_2020_1631_MOESM2_ESM.tif]

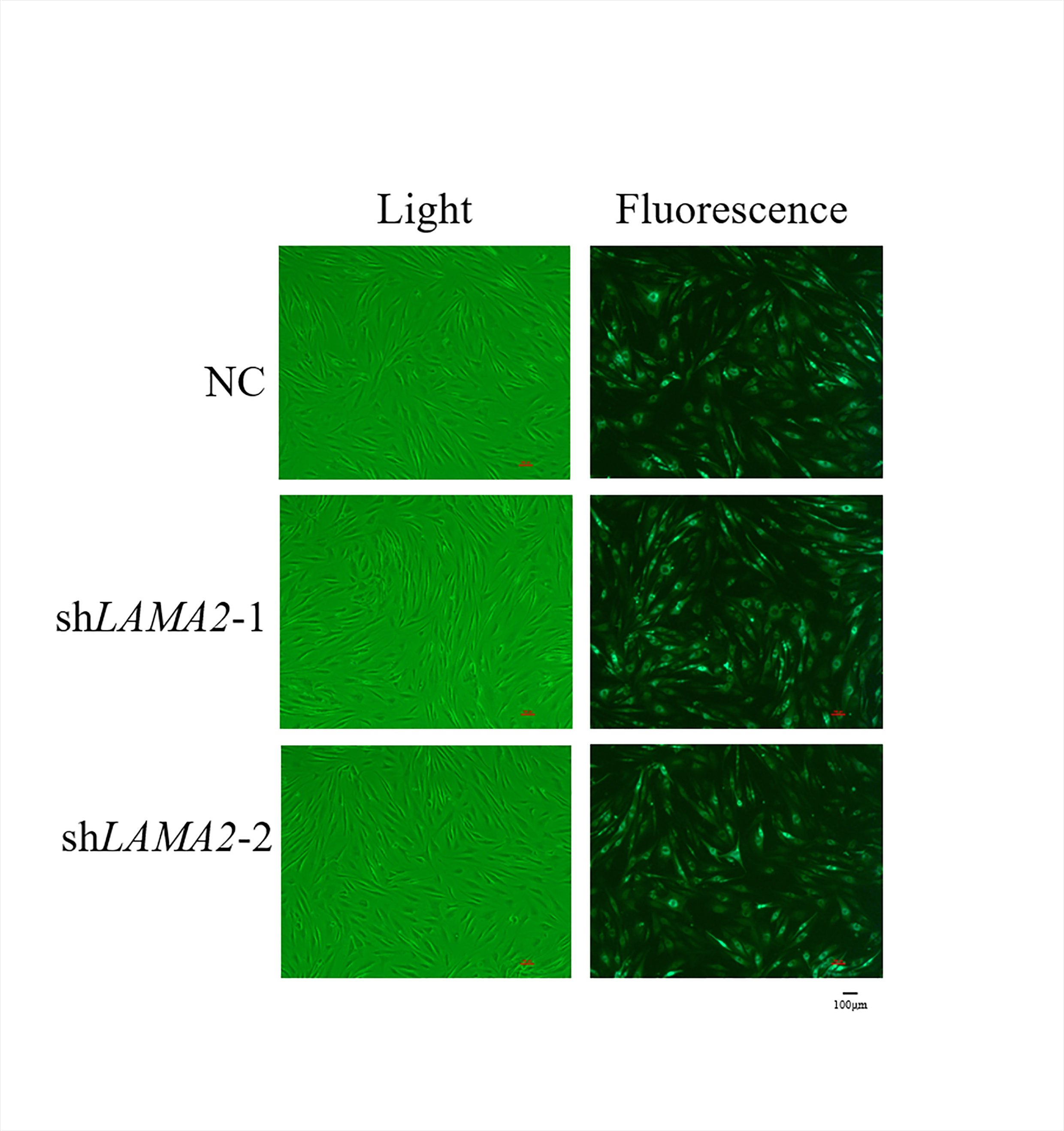

Supplement: Supplementary file 3 — Additional file 3: Figure S2. Microscopic images of green fluorescence protein (GFP)-positive MSCs under ordinary and fluorescent light. [file 13287_2020_1631_MOESM3_ESM.tif]

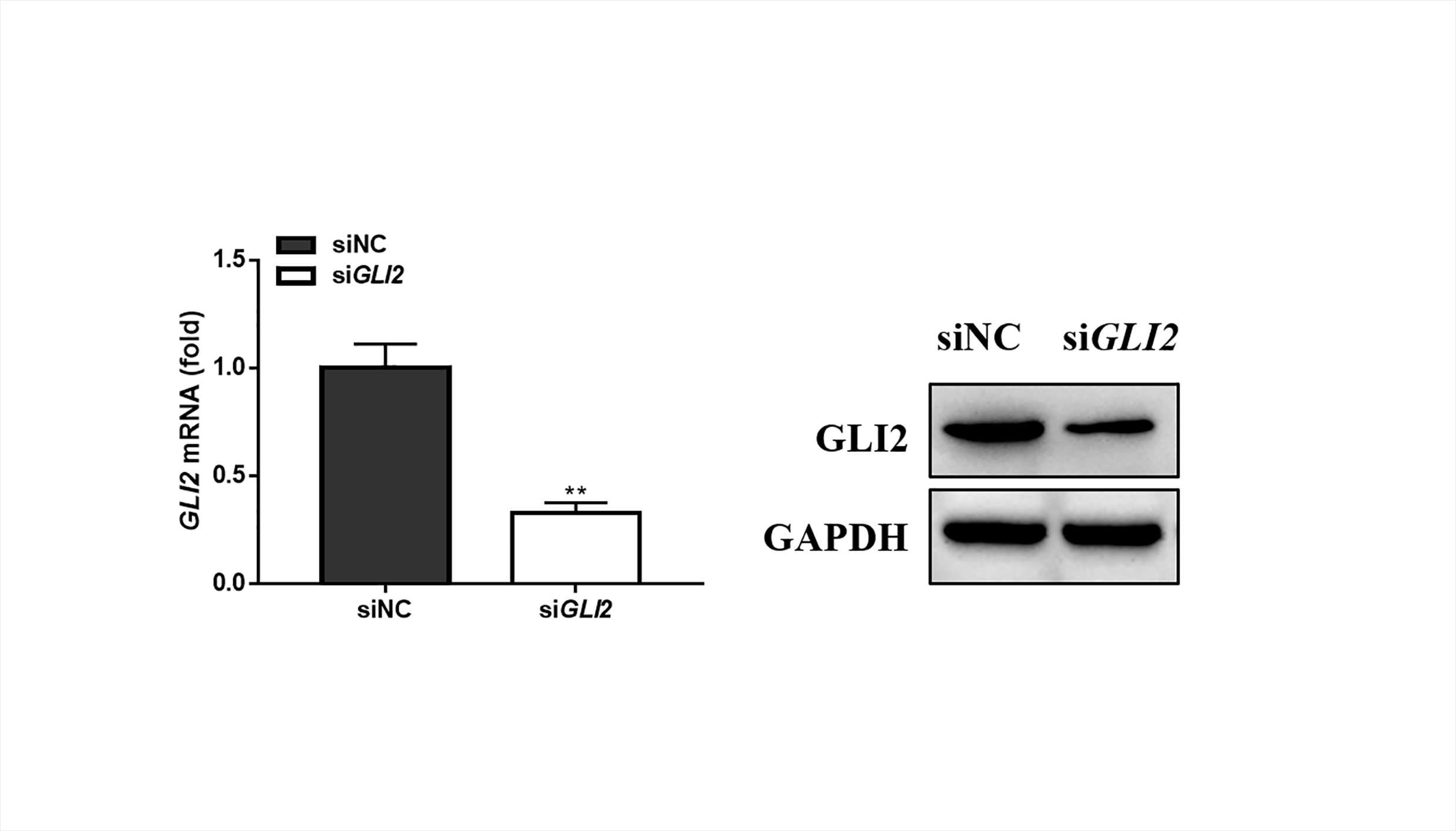

Supplement: Supplementary file 4 — Additional file 4: Figure S3. RT-qPCR and Western blot analysis were used to verify the efficiency of GLI2 silencing in MSCs. [file 13287_2020_1631_MOESM4_ESM.tif]
